# Stress-Related Regulation Is Abnormal in the Psoriatic Uninvolved Skin

**DOI:** 10.3390/life11070599

**Published:** 2021-06-23

**Authors:** Renáta Bozó, Judit Danis, Lili Borbála Flink, Dániel László Vidács, Lajos Kemény, Zsuzsanna Bata-Csörgő

**Affiliations:** 1 Department of Dermatology and Allergology, University of Szeged, 6720 Szeged, Hungary; danis.judit@med.u-szeged.hu (J.D.); flink.lili.borbala@med.u-szeged.hu (L.B.F.); vdani28@gmail.com (D.L.V.); kemeny.lajos@med.u-szeged.hu (L.K.); bata.zsuzsa@med.u-szeged.hu (Z.B.-C.); 2 HCEMM-USZ Skin Research Group, 6720 Szeged, Hungary; 3 MTA-SZTE Dermatological Research Group, Eötvös Loránd Research Network, 6720 Szeged, Hungary

**Keywords:** cell-stress, psoriasis, uninvolved skin, FOXO-mediated transcription, p27/CDKN1B

## Abstract

Keratinocyte stress-response of the uninvolved psoriatic epidermis is known to be altered compared to healthy cells. Therefore, we aimed to reveal potential mechanisms underlying this alteration. We compared the expression of annotated cell-stress-related proteins between uninvolved psoriatic and healthy skin using the protein array method. Data were analyzed by the Reactome over-representation test. We found that p27/CDKN1B and cytochrome C showed at least a two-fold increase, while cyclooxygenase-2, indolamine-2,3-dioxygenase-1, serum paraoxonase 1, serum paraoxonase 3, serine-46-phosphorylated tumor protein p53, and superoxide-dismutase-2 showed a two-fold decrease in expression in the uninvolved skin. Over-representation analysis suggested the Forkhead-box protein O (FOXO)-mediated transcription as the most significant pathway affected by the differently expressed cell-stress-related proteins (DECSRPs). DECSRPs indicate increased FOXO-mediated transcription of cell-cycle genes and reduced interleukin-signaling in the psoriatic uninvolved skin. Nuclear positivity of the FOXO-signaling-related p27/CDKN1B and FOXO1 are negatively correlated with the disease severity and showed increased expression in the uninvolved epidermis and also in healthy primary keratinocytes, which were grown on cartilage oligomeric matrix protein-coated surfaces. Our results indicate a cell-cycle inhibitory process, as a stress-related compensatory mechanism in the uninvolved epidermis, that could be responsible for blocking keratinocyte hyperproliferation in the psoriatic uninvolved skin, thus maintaining the symptomless skin phenotype.

## 1. Introduction

Chronic plaque-type psoriasis is the most common form of the immune-mediated inflammatory skin disease, psoriasis. Supposedly, genetic and environmental factors interplay in triggering the appearance of the characteristic red and silver scaly plaques on the skin of patients [1,2]. The pathomechanism of this multifactorial disease is not fully understood and with current therapeutic possibilities, recurrence of symptoms cannot yet be prevented [3]. It is noteworthy that in psoriasis the healthy-looking uninvolved skin has several cellular and extracellular alterations that make the skin susceptible to the development of symptoms [4]. Therefore, the psoriatic uninvolved skin of patients provides tissue samples where we can search for alterations relative to healthy skin. Abnormalities detected in symptom-free uninvolved skin could help us to better understand the development of the lesions. Structural differences have been previously described in the dermal–epidermal junction (DEJ) region of the uninvolved psoriatic skin. Several studies have described incomplete basement membrane below the basal keratinocyte layer of the epidermis [5,6,7] along with altered integrin expression of basal keratinocytes [8]. Moreover, increased expression of extracellular matrix components, such as fibronectin splice variant extra A domain-containing EDA^+^FN [9,10,11] and the cartilage oligomeric matrix protein were observed at the DEJ region of uninvolved skin. We have previously shown that these proteins affect keratinocyte proliferation [12]. Although it has been shown that keratinocytes from uninvolved skin are more sensitive to proliferative signals [13,14] and stress effects [15], there is no detectable keratinocyte hyperproliferation in the psoriatic uninvolved skin. 

Among several environmental factors that can influence the development of psoriatic disease, stress proved to be a determining one. One of the first pieces of evidence for an association between stress and induction of psoriatic symptoms is the Köbner phenomenon, which has been described in psoriasis. According to this long-known observation, the onset of psoriatic lesions in the uninvolved skin of psoriatic patients is more frequent after skin trauma [16]. It is also long-known that areas of the skin with more exposure to mechanical stress are more likely to develop psoriatic symptoms, such as elbows and knees [17]. The so-called tape-stripping method is frequently used for mechanical stress induction. Tape stripping increases the level of epidermal proliferation and transforming growth factor-alpha production in psoriatic uninvolved skin compared to healthy skin [18]. Furthermore, tape-stripping treatment that increases proteins related to wound healing (such as α5-integrin, EDA^+^FN, keratinocyte growth factor (KGF), as well as keratinocyte growth factor receptor/fibroblast growth factor receptor 2 (KGFR/FGFR2) in normal skin does not affect uninvolved skin. These proteins are already overexpressed without tape stripping on the psoriatic uninvolved skin [11]. Another sign of altered stress level in the uninvolved psoriatic skin is the elevated expression of the psoriasis susceptibility-related RNA gene induced by stress (PRINS), a long noncoding RNA with a potential role in stress response and downregulation of inflammation in keratinocytes [19,20,21].

So far, no comprehensive study has been available on the expression of the different stress-related proteins in the psoriatic uninvolved skin. Therefore, we aimed to compare the expression of annotated cell-stress-related proteins (CSRPs) between healthy and psoriatic uninvolved skin to better understand the mechanisms underlying the altered stress response of the psoriatic uninvolved skin, and to identify key stress-related pathways that may dominate in the psoriatic uninvolved skin. We found eight differentially expressed cell-stress-related proteins (DECSRPs) with two-fold changes in the psoriatic uninvolved skin compared to healthy skin. The most significant pathway affected by the DECSRPs was the Forkhead box protein O (FOXO)-mediated transcription. Moreover, analysis of the DECSRPs suggested enhanced expression of FOXO-mediated transcription of cell-cycle genes and decreased interleukin-signaling. Furthermore, we observed an enhanced, disease-severity-related nuclear positivity of the p27/CDKN1B and FOXO1 in the keratinocytes of psoriatic uninvolved epidermis, indicating a key role of these proteins in cell-cycle regulation. 

## 2. Materials and Methods

### 2.1. Skin Samples and Ethics

Skin samples (punch biopsies, diameter = 6 mm) were obtained from healthy volunteers (*n* = 10, age 30–65 years) and the uninvolved skin of patients with mild to moderate chronic plaque-type psoriasis (*n* = 7, age: 30–65 years, minimum of 6 cm from the involved region, PASI: 4.2–19). Psoriatic patients did not receive systemic therapy for at least 8 weeks and local therapy for at least 4 weeks. The study was approved by the Human Investigation Review Board of the University of Szeged (PSO-EDAFN-002, 34/2015, 3517, 23 February 2015, Szeged, Hungary) Tissue samples were collected after written informed consent, in accordance with the Helsinki Declaration. 

### 2.2. Protein Isolation from Skin Biopsies

For the preparation of skin tissue protein extracts from each different donor (healthy volunteers *n* = 4, psoriatic uninvolved patients *n* = 4), skin-punch biopsies were homogenized in phosphate-buffered saline in the presence of 1% protease inhibitor cocktail (Sigma-Aldrich, St. Louis, MO, USA) and phenylmethylsulphonyl fluoride (Sigma Aldrich, St. Louis, MO, USA). After homogenization, Triton X-100 (Sigma Aldrich, St. Louis, MO, USA) was added to the homogenates to a final concentration of 1% and a cycle of freezing (at −80 °C) and thawing (at room temperature) was performed. Homogenates were separated by centrifugation and the total protein concentration of the supernatants was measured by bicinchoninic acid (BCA, Thermo Fisher Scientific, Waltham, MA, USA) assay. 

### 2.3. Cell-Stress Protein Array

Healthy and psoriatic uninvolved protein pools were prepared using the same amount of protein (50 µg) from the appropriate protein extracts. From these pooled protein extracts, the level of cell-stress-related proteins was determined by applying a human proteome profiler cell-stress array kit (R&D Systems, Minneapolis, MN, USA) according to the manufacturer’s instructions. A total of 26 different cell-stress-related proteins (ADAMTS1: a disintegrin-like metalloprotease with thrombospondin Type 1 Motif, 1; BCL2: apoptosis regulator Bcl-2; CA9: carbonic anhydrase 9; CITED2: Cbp/P300 interacting transactivator 2; COX2/PTGS2: cyclooxigenase-2; CYCS: cytochrome C; DKK4: Dickkopf WNT signaling pathway inhibitor 4; FABP1: fatty acid binding protein 1; HIF1A: hypoxia-inducible factor 1 subunit alpha; HIF2A: hypoxia-inducible factor 2-alpha; p-HSP27 (S78/S82): S78/S82 phosphorylated heat shock 27 kDa protein 1; HSP60: 60 kDa heat shock protein, mitochondrial; HSP70: 70 kDa heat shock protein; IDO1: indoleamine 2,3-dioxygenase 1; phospho-JNK Pan (T183/Y185): T183/Y185 phosphorylated mitogen-activated protein kinase 8; NFKB1: nuclear factor kappa B subunit 1; p21/CDKN1A: cyclin-dependent kinase inhibitor 1A; p27/CDKN1B: cyclin-dependent kinase inhibitor 1B; p-p38α (T180/Y182): T180/Y182 phosphorylated p38 mitogen-activated protein kinase; p-p53 (S46): S46 phosphorylated tumor protein p53; PON1: serum paraoxonase 1; PON2: serum paraoxonase 2; PON3: serum paraoxonase 3; TXN: thioredoxin-1; SIRT2: sirtuin-2; and SOD2: superoxide-dismutase 2) can be detected in duplicates with this method. Signal was visualized on a C-Digit blot scanner (LI-COR Biosciences, Lincoln, NE, USA) with Clarity Max ECL substrate (Bio-Rad Laboratories, Hercules, CA, USA). 

### 2.4. Data Analysis

Pixel densities of the spots were collected by Image Studio software (LI-COR Biosciences, Lincoln, NE, USA), and the average pixel densities of the pairs of the duplicate spots were determined, representing each cell-stress-related protein. The average background signal was subtracted from each spot and the corresponding signals on different array membranes (healthy and psoriatic uninvolved) were compared. The relative changes in the expression of cell-stress-related proteins were determined. Only proteins showing a two-fold increase or two-fold decrease were determined as differentially expressed cell-stress-related proteins (DECSRPs). 

The publicly available Reactome database (https://reactome.org, accessed on 12 October 2020) was applied to perform an over-representation analysis using the analysis tool of the Reactome database to identify biological processes and pathways that were overrepresented in our dataset. Each gene identifier of the DECSRPs was uploaded and mapped to the corresponding gene object in the database. Possible affected biological processes and pathways were identified by the software. The type of Reactome over-representation analysis was a gene list analysis that was projected to the human organism. Results were filtered by statistical significance (*p* ≤ 0.05).

### 2.5. Immunofluorescence Staining

Healthy and psoriatic uninvolved skin biopsies (*n* = 3) were paraffin-embedded and cut into 5-μm thick sections. After heat-recovery (in citrate buffer, pH = 6), samples were fixed in 4% paraformaldehyde (PFA) and permeabilized in 0.25% Triton X-100 (Thermo Fisher Scientific, Waltham, MA, USA) solution. For blocking, 1% bovine serum albumin (Sigma Aldrich, St. Louis, MO, USA) and 1% normal goat serum (Sigma Aldrich, St. Louis, MO, USA) were used. Samples were incubated overnight at 4 °C with primary monoclonal mouse antihuman p27/CDKN1B and FOXO1 antibodies (dilution: 1:100, (BioLegend, San Diego, CA, USA). Mouse IgG1 (Beckton Dickinson, Franklin Lakes, NJ, USA) served as isotype control. As a secondary antibody, Alexa Fluor 647-conjugated antimouse IgG (Life Technologies, Carlsbad, CA, USA) was used (dilution: 1:500). Nuclei were marked by 4′,6-diamidino-2-phenylindole (DAPI) (Sigma Aldrich, St. Louis, MO, USA) staining. Zeiss Axio Imager Z1 microscope (Carl Zeiss AG, Oberkochen, Germany) was applied for visualization. To determine the nuclear positivity of p27/CDKN1B in the epidermis, three randomly selected areas were selected per donor, where cells with p27/CDKN1B nuclear positivity were calculated. Two-tailed Student *t*-test was performed to detect statistical differences using R-Studio software, version 3.2.2 (R-Studio, Boston, MA, USA). * *p* ≤ 0.05 was considered statistically significant.

### 2.6. Examination of p27/CDKN1B and FOXO1 Localization in Normal Human Epidermal Keratinocytes (Nheks)

NHEK cells were isolated and cultured from the healthy skin samples as previously described [11,22]. Briefly, 6 mm punch biopsy skin specimens were incubated in Dispase (Roche Diagnostics, Basel, Switzerland) at 4 °C overnight; subsequently, the dermis and epidermis were separated. To isolate keratinocytes, the epidermis was incubated in Trypsin-EDTA solution (Sigma Aldrich, St. Louis, MO, USA) for 5 min, washed in sterile PBS, and cultured in keratinocyte SFM supplemented with epidermal growth factor, brain pituitary extract (Life Technologies, Carlsbad, CA, USA), and 1% L-glutamine and 1% antibiotic/antimycotic solution (Sigma Aldrich, St. Louis, MO, USA). Cells were cultured at 37 °C in a humidified atmosphere with 5% *v*/*v* CO_2_. NHEK cells were plated onto an 8-well chamber slide at a density of 20,000 cells per well, where the surface was coated with 10 μg/mL recombinant human COMP protein (R&D Systems, Minneapolis, MN, USA), [12] or with 3,2 μg/mL fibronectin protein (from bovine plasma, Sigma Aldrich, St. Louis, MO, USA). The uncoated surface served as a control. After 72 h post-seeding, cells were fixed with 4% PFA, and were immunolabeled with p27/CDKN1B and FOXO1 antibodies as above described. Five randomly selected areas were selected per group, where total cell numbers and cells with p27/CDKN1B and FOXO1 nuclear positivity were calculated. One-way ANOVA with the Tukey posthoc test was used to detect statistical differences using R-Studio software, version 3.2.2 (R-Studio, Boston, MA, USA). * *p* ≤ 0.05 was considered statistically significant.

## 3. Results

### 3.1. Annotated Cell-Stress-Related Proteins (CSRPs) Are Expressed in Healthy Aand Psoriatic Uninvolved Skin

**Figure 1:**
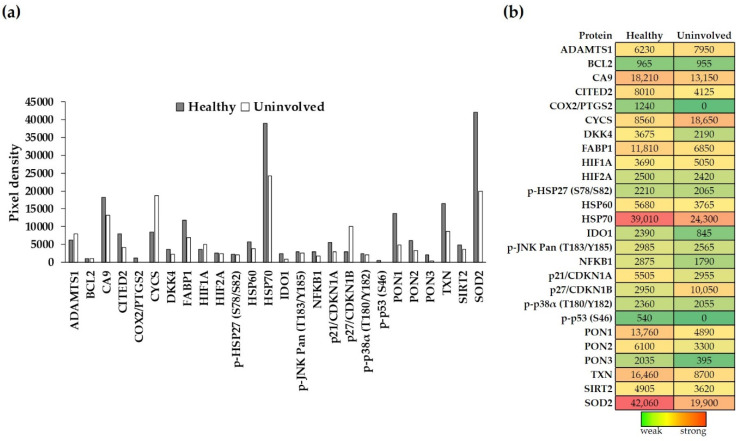
Expression of cell-stress-related proteins in the healthy and psoriatic uninvolved skin. (**a**,**b**) Cell-stress-related protein (CSRP) expression in the healthy (gray, *n =* 4, pooled protein extract) and psoriatic uninvolved (white, *n* = 4, pooled protein extract) whole-skin biopsies were determined by a cell-stress protein array. Pixel densities of the different proteins representing their expression levels are presented. Pixel densities were determined by the Image Studio software (LI-COR Biosciences, Lincoln, NE, USA). Green color indicates the lowest, red color the highest, and yellow color indicates medium expression of the given protein in the skin. Colors also reflect the differences between healthy and psoriatic uninvolved skin. (CSRPs indicated by gene identifiers: ADAMTS1: a disintegrin-like metalloprotease with thrombospondin Type 1 Motif, 1; BCL2: apoptosis regulator Bcl-2; CA9: carbonic anhydrase 9; CITED2: Cbp/P300 interacting transactivator 2; COX2/PTGS2: cyclooxigenase-2; CYCS: cytochrome C; DKK4: Dickkopf WNT signaling pathway inhibitor 4; FABP1: fatty acid binding protein 1; HIF1A: hypoxia-inducible factor 1 subunit alpha; HIF2A: hypoxia-inducible factor 2-alpha; p-HSP27 (S78/S82): S78/S82 phosphorylated heat shock 27 kDa protein 1; HSP60: 60 kDa heat shock protein; HSP70: 70 kDa heat shock protein; IDO1: indoleamine 2,3-dioxygenase 1; phospho-JNK Pan (T183/Y185): T183/Y185 phosphorylated mitogen-activated protein kinase 8; NFKB1: nuclear factor kappa B subunit 1; p21/CDKN1A: cyclin-dependent kinase inhibitor 1A; p27/CDKN1B: cyclin-dependent kinase inhibitor 1B; p-p38α (T180/Y182): T180/Y182 phosphorylated p38 mitogen-activated protein kinase; p-p53 (S46): S46 phosphorylated tumor protein p53; PON1: serum paraoxonase 1; PON2: serum paraoxonase 2; PON3: serum paraoxonase 3; TXN: thioredoxin-1; SIRT2: sirtuin-2; and SOD2: superoxide-dismutase 2).

To gain a deeper understanding of the possible stress-induced changes in psoriatic uninvolved skin, we applied a cell-stress protein array on whole-skin biopsies of healthy and uninvolved skin samples. The array allows simultaneous detection of numerous proteins from the same sample. The expression level of cell-stress proteins was compared between healthy and psoriatic uninvolved whole skin biopsies (Figure 1a). All 26 investigated cell-stress proteins were detectable in the healthy skin, namely: ADAMTS1; BCL2; CA9; CITED2; COX2/PTGS2; CYCS; DKK4; FABP1; HIF1A; HIF2A; p-HSP27 (S78/S82); HSP60; HSP70; IDO1; phospho-JNK Pan (T183/Y185); NFKB1; p21/CDKN1A; p27/CDKN1B; p-p38α (T180/Y182); p-p53 (S46); PON1; PON2; PON3; TXN; SIRT2; and SOD2. In contrast, only 24 out of the 26 proteins were detectable in the psoriatic uninvolved skin. COX2/PTGS2 and p-p53 (S46) were undetectable in the uninvolved skin (Figure 1b). 

### 3.2. The Expression of Cell-Stress-Related Proteins in Psoriatic Uninvolved Skin Is Altered in Comparison with Healthy Skin

**Figure 2:**
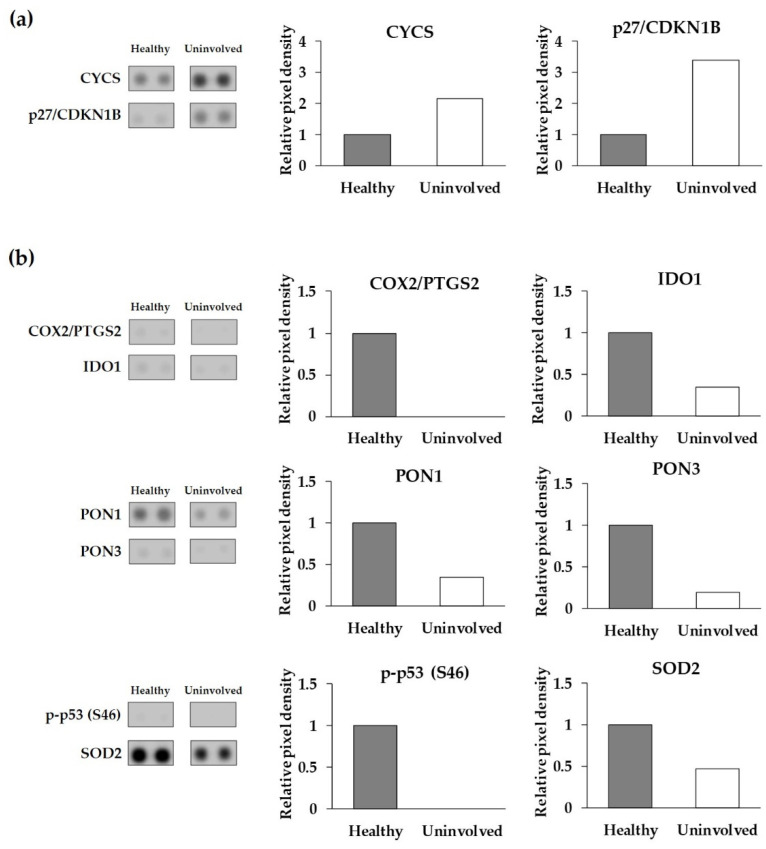
Differentially expressed cell-stress-related proteins in the psoriatic uninvolved skin. Cell-stress protein array spots and relative pixel density of the differentially expressed cell-stress-related proteins (**a**) with a two-fold increase and (**b**) with a two-fold decrease in the psoriatic uninvolved skin. Data are presented as relative, healthy normalized pixel density (*n* = 4, pooled healthy and psoriatic uninvolved protein extracts). Pixel densities were determined by the Image Studio software (LI-COR Biosciences, Lincoln, NE, USA). DECSRPs are indicated by gene identifiers: CYCS: cytochrome C; p27/CDKN1B: cyclin-dependent kinase inhibitor 1B; COX2/PTGS2: cyclooxygenase-2; IDO1: indoleamine 2,3-dioxygenase 1; PON1: serum paraoxonase 1; PON3: serum paraoxonase 3; p-p53 (S46): S46 phosphorylated tumor protein p53; SOD2: superoxide-dismutase 2.

Differences were observed not only in the number of CSRPs expressed in the psoriatic uninvolved skin but also in their expression patterns. Although some proteins had similar expression levels in healthy and psoriatic uninvolved skin, most of them showed different expressions. Proteins with at least a two-fold increase or two-fold decrease between healthy and psoriatic uninvolved skin were selected as differentially expressed cell-stress-related proteins (DECSRPs). Based on the measured pixel densities, 8 out of the 26 CSRPs, namely, CYCS, p27/CDKN1B, COX2/PTGS2, IDO1, PON1, PON3, p-p53 (S46), and SOD2 showed at least two-fold changes in psoriatic uninvolved skin samples compared to healthy skin samples (Figure 2). These proteins were determined as DECSRPs. Compared to healthy skin, the expression level of the CYCS and p27/CDKN1B were increased (Figure 2a), while, COX2/PTGS2, IDO1, PON1, PON3, p-p53 (S46), and SOD2 were decreased in the psoriatic uninvolved skin (Figure 2b), indicating altered stress-related processes in the psoriatic uninvolved skin. 

### 3.3. The FOXO-Mediated Transcription Is Over-Represented by the Differentially Expressed Cell-Stress-Related Proteins in the Psoriatic Uninvolved Tissue

**Figure 3:**
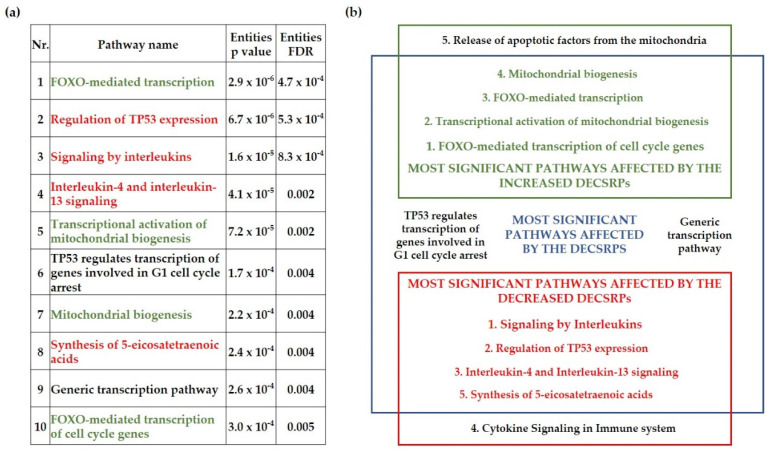
Biological pathways influenced by the differentially expressed cell-stress-related proteins in the psoriatic uninvolved skin. The most significant pathways affected by differentially expressed cell-stress-related proteins (DECSRPs) (**a**) and by the DECSRPs with a two-fold increase or decrease (**b**) were determined by using the Reactome over-representation analysis tool (www.reactome.org, accessed on 12 October 2020). Results were filtered by statistical significance (*p* ≤ 0.05). Green color indicates pathways affected by DECSRPs showing two-fold increased expression, red color marks pathways that are affected by the DECSRPs with two-fold decreased expression. Numbering indicates the order of significance obtained during the over-representation analysis.

To gain insight into the biological pathways affected by the DECSRPs, Reactome over-representation analysis was applied. The 10 most significant pathways affected by the DECSRPs were presented in Figure 3a. Eight out of the 10 most significant pathways affected by the CSRPs can be associated with the known pathogenesis of psoriasis, suggesting a strong relationship between the DECSRPs and psoriasis. Interestingly, the FOXO-mediated transcription was determined as the most significant pathway affected by the DECSRPs (Figure 3a). Moreover, the FOXO-mediated transcription of the cell-cycle genes was the highest rank pathway influenced by DECSRPs showing increased expression, indicating enhanced FOXO-mediated transcription in psoriatic uninvolved skin. Furthermore, as the over-representation analysis suggests, DECSRPs with increased expression are also linked to enhanced mitochondrial biogenesis and enhanced release of apoptotic factors from the mitochondria (Figure 3b). DECSRPs with decreased expression mostly influence pathways of interleukin (IL) signaling, according to the over-representation analysis, suggesting impaired IL-signaling in the psoriatic uninvolved skin. Moreover, based on DECSRPs with two-fold decrease, the most affected pathways were the regulation of TP53 expression, the IL-4 and IL-13 signaling, the synthesis of 5-eicosatetraenoic acids, and cytokine signaling in the immune system (Figure 3b), indicating altered immune and fatty acid homeostasis in the psoriatic uninvolved skin.

### 3.4. Nuclear Expression of p27/CDKN1B in the Psoriatic Uninvolved Epidermis Is Increased

**Figure 4:**
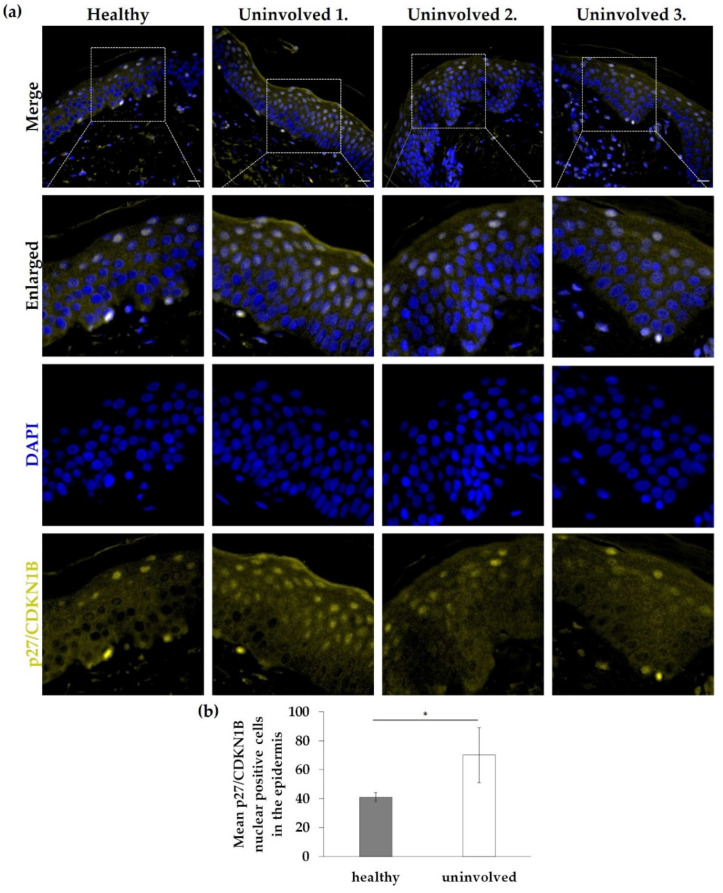
Immunolocalization of p27/CDKN1B in skin samples from healthy and psoriatic uninvolved skin. (**a**) Tissue expression of p27/CDKN1B in the skin specimens obtained from healthy donors (*n* = 3, the representative image is shown) and from uninvolved skin of patients with mild to moderate psoriasis (*n* = 3, PASI scores: Uninvolved 1.: 4.2; Uninvolved 2.: 13.8; Uninvolved 3.: 17). DAPI: 4′,6-diamidino-2-phenylindole; Zeiss AxioImager Z1 microscope, 40× original magnification, scale bar: 20 μm. (**b**) The mean p27/CDKN1B nuclear positive cell number in the epidermis was calculated. The graph shows mean +/− SD (*n* = 3 donors with three randomly selected areas of each group). * *p* ≤ 0.05, calculated by two-tailed Student t-test.

The FOXO-mediated transcription and FOXO-mediated transcription of cell cycle genes proved to be a key mechanism influenced by DECSRPs with increased expression in psoriatic uninvolved skin samples. p27/CDKN1B, one of the identified increased DECSRPs, is a downstream element of the FOXO-mediated transcription, linked to cell-cycle regulation. To examine p27/CDKN1B expression and localization, immunofluorescence staining was used on healthy skin and uninvolved skin from patients with mild to moderate psoriasis. In healthy skin, p27/CDKN1B expression was mainly cytoplasmic and nuclear positivity was not obvious, while in psoriatic uninvolved skin enhanced nuclear positivity of p27/CDKN1B (Figure 4a,b) was detected mainly in the uppermost layers of the psoriatic uninvolved epidermis. Mean nuclear positivity of p27/CDKN1B was significantly increased in the uninvolved epidermis (Figure 4b). Interestingly, the highest nuclear positivity was found in the epidermis of a psoriatic patient with the lowest PASI score (Figure 4a). 

### 3.5. The FOXO1 Expression Pattern in the Psoriatic Uninvolved Skin Is Altered Compared to Normal Skin

**Figure 5:**
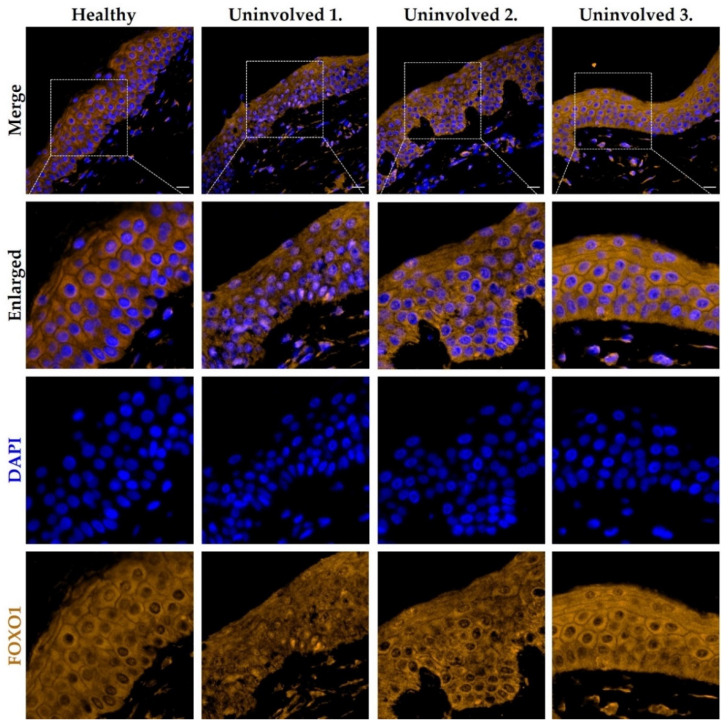
Immunolocalization of FOXO1 in skin samples from healthy and psoriatic uninvolved skin. Tissue expression of FOXO1 in the skin samples obtained from healthy donors (*n* = 3, the representative image is shown) and from uninvolved skin of patients with mild to moderate psoriasis (*n* = 3, PASI scores: Uninvolved 1.: 4.2; Uninvolved 2.: 13.8; Uninvolved 3.: 17). DAPI: 4′,6-diamidino-2-phenylindole; Zeiss AxioImager Z1 microscope, 40× original magnification, scale bar: 20 μm.

To further examine the FOXO-mediated signaling in the psoriatic uninvolved skin, FOXO1 immunofluorescence staining was applied on healthy and on psoriatic uninvolved skin. In the healthy control samples, FOXO1 was mainly localized in the keratinocyte cytoplasm with a homogenous staining pattern. In the uninvolved epidermis of patients with lower PASI score, the nuclear positivity of the FOXO1 was enhanced and the cytoplasmic expression pattern was altered compared to healthy skin. Interestingly, the uninvolved skin of the psoriatic patient with the lowest PASI scores showed the most elevated nuclear positivity of FOXO1, similar to the p27/CDKN1B expression. In the uninvolved epidermis of patients with higher PASI scores, a similar cytoplasmic homogeneous staining pattern to that of healthy epidermis was observed (Figure 5). The p27/CDKN1B and FOXO1 immunofluorescence staining results suggest an altered, disease-severity correlated cell-cycle regulation in the psoriatic uninvolved epidermis. 

### 3.6. Nuclear Localization of P27/CDKN1B and FOXO1 Was Elevated in Normal Human Epidermal Keratinocytes (NHEKs) Cultured On COMP-Coated Surfaces

**Figure 6:**
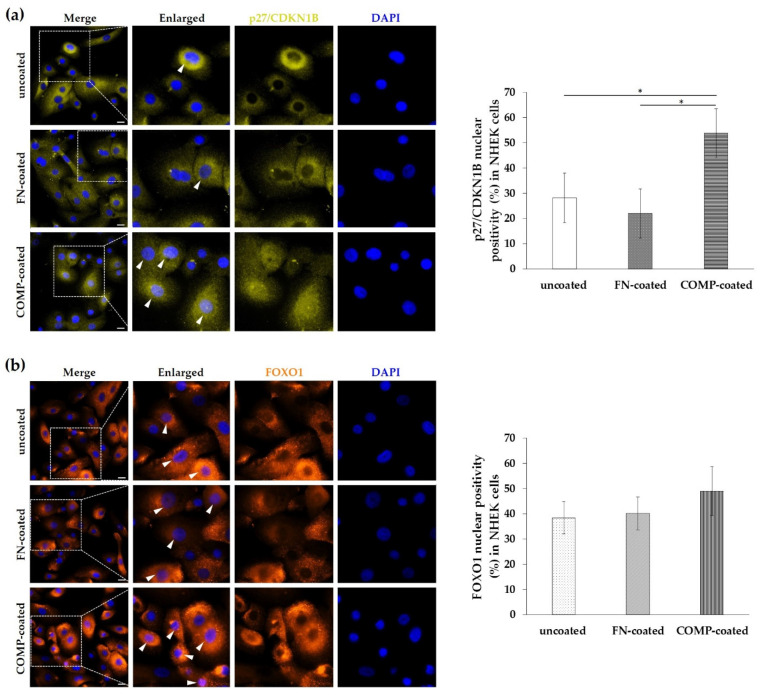
Immunolocalization of p27/CDKN1B and FOXO1 in healthy primary keratinocytes. Normal human epidermal keratinocytes (NHEKs) were cultured on uncoated, fibronectin (FN)-coated, and cartilage oligomeric matrix protein (COMP)-coated surfaces. Cellular expression of (**a**) p27/CDKN1B and (**b**) FOXO1 was examined with immunofluorescence staining. Representative images are shown from three independent experiments. Dotted lines indicate the enlarged regions, arrowheads mark the positive nuclei. DAPI: 4′,6-diamidino-2-phenylindole; Zeiss AxioImager Z1 microscope, 40× original magnification, scale bar: 20 μm. The mean nuclear positivity ratio (%) of p27/CDKN1B and FOXO1 was calculated. The graph shows mean +/− SD (*n* = 3 biological replicates with five randomly selected areas of each group). * *p* ≤ 0.05 calculated by one-way ANOVA followed by Tukey’s posthoc test.

We know that p27/CDKN1B and FOXO1 are involved in the reduction of the cell proliferation. In our previous studies, COMP proved to reduce the proliferation rate of keratinocytes, therefore we examined the localization of p27/CDKN1B and FOXO1 in NHEK cells cultured on uncoated or COMP- or fibronectin-coated surfaces to reveal a potential regulatory relationship. We found that COMP coating resulted in a tendentious increase in FOXO1 nuclear positivity in NHEKs (Figure 6b) and a significantly higher nuclear positivity of p27/CDKN1B (Figure 6a).

## 4. Discussion

An increasing body of evidence suggests that altered stress response in psoriasis plays an important role in the pathogenesis of the disease. The PRINS long noncoding RNA was first identified by our research group with the highest expression in the psoriatic uninvolved epidermis compared to healthy and lesional psoriatic skin [19]. It was revealed that PRINS can alter the stress response of uninvolved keratinocytes contributing the disease pathogenesis. The expression of PRINS was inducible with several stress factors indicating different stress-related mechanisms in the psoriatic uninvolved skin, where PRINS is overexpressed [21]. It has long been known that keratinocytes of the uninvolved epidermis are more sensitive to stress [15] and their sensibility for proliferative signals is enhanced [13,14]. Hence, investigation of potential factors and mechanisms characterizing the uninvolved skin can bring us closer to understanding how the tissue environment can affect the appearance of psoriatic plaques or help to maintain the symptomless phenotype of the uninvolved skin. 

Regulation of the stress response can take place at different levels; therefore, we performed a cell-stress protein array to widely reveal regulation of the stress response in uninvolved skin compared to healthy skin at the protein level. We found that all the investigated CSRPs are expressed in the healthy skin, and 24 out of the 26 CSRPs were also detectable in the psoriatic uninvolved skin. Moreover, most of the examined proteins were differentially expressed in the psoriatic uninvolved skin compared to healthy skin. Among the investigated CSRPs, proteins with two-fold changes were selected and further analyzed. By using the Reactome over-representation test, suitable for the determination of pathways that are enriched in the analyzed datasets, we identified the FOXO-mediated transcription as the most significantly affected by the DECSRPs. The FOXO transcription factor family is responsible for the regulation of cell proliferation and survival [23], processes known to be crucial in the pathogenesis of psoriasis. Activation of FOXO leads to inhibition of cell proliferation and promotion of the quiescent state of the cells [24]. In psoriatic involved skin, FOXO1 is down-regulated compared to the healthy and uninvolved skin as well, but interestingly, it is reactivated following etanercept treatment [25]. 

Analysis of only the increased DECSRPs using the same over-representation test identified the FOXO-mediated transcription of the cell cycle, as the most significantly altered pathway in the uninvolved psoriatic skin, suggesting increased cell cycle inhibition. Besides the FOXO-mediated transcription, this analysis revealed that mitochondrial biogenesis and functions were also altered in the uninvolved skin. There is evidence that mitochondrial dysfunction contributes to reduced keratinocyte apoptosis in psoriatic involved skin with reduced expression of the mitochondrial function regulator genes (uncoupling protein 2, dynamin-related protein 1, and calcineurin) [26]. Further evidence is required to fully answer the question of how mitochondrial functions influence the uninvolved skin. 

The most significant pathway affected by decreased DECSRPs was the interleukin signaling based on the over-representation test. Out of the 5 most affected processes, 3 can be related to cytokine/interleukin signaling. Data indicate that these processes are disturbed, less active in the psoriatic uninvolved skin in comparison with healthy skin suggesting anti-inflammatory processes. The analysis of the decreased DECSRPs also suggested an altered, less active fatty-acid metabolism and TP53-dependent transcription to be present in the uninvolved skin. These processes have long been indicated as players in the pathogenesis of psoriasis [27,28].

Since the analysis indicated the FOXO-mediated processes as key mechanisms, and this pathway is not fully examined in uninvolved skin, we further investigated this pathway. p27/CDKN1B is a downstream element of the FOXO-signaling, and a negative regulator of cell-cycle progression due to inhibition of G1/S transition [29]. It has been reported that in lesional psoriatic skin p27/CDKN1B expression is reduced in keratinocytes, parallel with hyperproliferation. In that same study p27/CDKN1B showed decreased expression in nonlesional epidermis, but, in some cases, immunolabeled nuclei for p27 in the uppermost keratinocytes were also observed [30]. Another study described that the expression of p27/CDKN1B was increased in the uninvolved skin compared to involved skin based on immunostaining [31]. In our present study, we detected enhanced nuclear positivity of the p27/CDKN1B in the upper layers of the psoriatic uninvolved epidermis and this nuclear positivity showed a negative correlation with disease severity. While in the previous study all investigated patients had PASI above 15, we obtained samples from patients with PASI scores between 4.2–17, allowing for comparison of patient samples with different severity. In parallel with the increased p27/CDKN1B expression in uninvolved skin samples of patients with low PASI scores, FOXO1 staining also showed increased nuclear positivity. FOXO1 is known to play a role in cell-cycle arrest through regulation of the nuclear localization of the p27/CDKN1B [24]. These results indicate that in the uninvolved epidermis of patients with lower PASI scores, a more active cell-cycle inhibition could be responsible for maintaining the nonhyperproliferative state of the keratinocytes. 

In a previous study, we reported, that COMP-coating resulted in a reduced proliferation rate of NHEK cells. Thus, increased expression of COMP at the DEJ of the uninvolved skin can restrain keratinocyte proliferation, contributing to the maintenance of the symptomless, nonhyperproliferative state [12]. Indeed, the present study revealed, that culturing keratinocytes on COMP-coated dishes results in a tendentious increase in nuclear positivity of FOXO1 and a significant increase in p27/CDKN1B expression in the cultured cells. These observations suggest that COMP at least in part can contribute to the FOXO1-p27/CDKN1B mediated inhibition of cell-cycle progression. 

Abnormal cell-cycle regulation and interleukin-driven inflammatory changes are well-known disease-contributing factors in psoriasis [3], and as our data show they are the most affected stress-related pathways in the uninvolved skin. It is possible that in psoriasis the entire skin has inherited the capacity to form lesions [32], and special compensatory mechanisms function to maintain the seemingly healthy-looking skin phenotype. These mechanisms are related to stress and they are connected to cell-cycle regulation and interleukin signaling. Uncovering the factors that are involved in protecting a healthy-looking skin phenotype in psoriasis could reveal potential new therapeutic targets, which may help to avoid the recurrence of the symptoms. 

## Data Availability

Not applicable.
